# Correction: Study on the releasing regularity of asphalt fume and its suppression technology

**DOI:** 10.1371/journal.pone.0336609

**Published:** 2025-11-10

**Authors:** Guang Yang, Yongli Xu, Xiaolei Jiao, Yiming Li

The authors, Yongli Xu and Yiming Li, should be noted as shared co-corresponding authors on this work. Yongli Xu’s email address is: xuyongli@nefu.edu.cn
