# Supplementary material for: Study on the releasing regularity of asphalt fume and its suppression technology
Source: PLoS One. 2025 Aug 4;20(8):e0329225. doi: 10.1371/journal.pone.0329225 (PMC12321099; doi:10.1371/journal.pone.0329225)
Supplement: Attachment — Submitted filename: Response to Reviewers.docx [file pone.0329225.s002.docx]

Dear Editors and Reviewers:

On behalf of my co-authors, we thank you very much for giving us an opportunity to revise our manuscript, we appreciate the reviewers very much for their positive and constructive comments and suggestions on our manuscript entitled "Study on the Releasing Regularity of Asphalt Fume and Its Suppression Technology". (Manuscript ID: PONE-D-25-06131). We have studied the comments carefully and made revision which we hope meet with approval.

The main corrections in the paper and the responds to the reviewer's comments are as flowing:

Response to the reviewers' comments:

**Reviewer #1:**

This manuscript is a study of the release patterns and inhibition techniques for asphalt fumes. Smoke-suppressing and deodorizing asphalt was prepared by adding SBS, MOS and deodorizer to the matrix asphalt. The smoke inhibition and deodorization of matrix asphalt by smoke inhibitor and deodorizer were also analyzed by mass method, odor intensity grading method, FTIR, SEM and TG-MS. However, the manuscript has more detailed problems, and the specific modifications are as follows:

**Comment 1:** There are some problems with the structure and logic of the introduction section. The introduction of the fume collection device seems rather abrupt and does not form an effective connection with the contextual content. In addition, several concluding statements lacked the support of corresponding literature citations.

**Reply 1:** We gratefully appreciate for your valuable suggestion. The relevant part of the introduction has been revised and the relevant references have been added.

**Comment 2:** The manuscript is inaccurate in its description of Table I. Is SBS a modifier or a bitumen? What is the purpose of mentioning different matrix bitumens in Table I? Were these asphalts set up as controls? The tensile speed for the ductility test should be 50 mm/min. In addition, the specifications for the modifier MOS and deodorizer need to be listed.

**Reply 2:** We appreciate your careful check and feel sorry that we didn't notice this clearly mistake. We have revised the table, and the specifications for the modifier MOS and deodorizer have been listed.

**Comment 3:** Is the use of PTFE filter membranes supported by the results of other studies?

**Reply 3:** The use of PTFE filter membranes can be found in the following documents: 1. Hao, Gengren. "Characterization and quantitative analysis of asphalt fumes generated in asphalt pavement materials." (2022). 2. Guo, M., Xu, W., & Du, X. (2024). Study on the release and suppression of fumes from asphalt binder. Construction and Building Materials, 441, 137511. The references have been added to the manuscript. Thanks for your comment.

**Comment 4:** The description of the test program in Table 2 is very vague, please redraw the table.

**Reply 4:** We agree with your valuable comment. This part was not written clearly and we have revised this part and added the experimental method.

**Comment 5:** In “2.2methods for asphalt fume”, please add the description of TG test and DSC test methods.

**Reply 5:** Thanks again for your valuable suggestions. Procedures of odour test, SEM and TG have been described in the sections 2.2.4, 2.2.5.

**Comment 6:** In subsection 3.1, the author's description of Figures 6 and 7 is wrong. The release of asphalt fume tends to increase with the increase of heating time and heating temperature.

**Reply 6:** We must apologize for this clearly mistake and thank you for your correction. The relevant part of the manuscript has been revised accordingly.

**Comment 7:** In the summary of the release pattern of asphalt smoke, the release of asphalt smoke is greater when the temperature is 180°C and the time is 5 h. Why were the test conditions finally determined at a heating temperature of 160°C and a time of 3 h?

**Reply 7:** In the following experiment, heating temperature and time are the control variables, so the choice doesn't affect the outcome of following experiment. Considering the actual processing conditions in asphalt pavement engineering, we choose this temperature and time in order to make the experimental results have practical significance. Relevant interpretation has been added to the manuscript.

**Comment** **8:** In Fig. 12, it is suggested to add obvious labeling to point out the location where the MOS whiskers are exposed

**Reply 8:** Thanks for your suggestions, we have added the labeling in the figure.

**Comment 9:** In both the FTIR and TG tests, the tests were carried out on five types of bitumen. Therefore, it is suggested that the SEM test, DSC test and TG-MS test should also be carried out on five kinds of bitumen to ensure the comprehensiveness of the test.

**Reply 9:** Thanks for your valuable suggestions, This study has determined the content of each part of the bitumen for purifying smoke suppression through odor testing and pavement performance experiments, focusing on the mechanism of purifying smoke suppression bitumen. We admit that it is indeed valuable to conduct a comparative test on five kinds of bitumen, but due to limited space and time, we plan to carry out this part in the subsequent study.

**Comment 10:** The road performance of modified asphalt is mentioned several times in the manuscript, but the results of the effect of various modifiers on the road performance of asphalt are not reflected in the whole text, so it is suggested that the authors add.

**Reply 10:** Thanks for your advice. We have added the road performance of asphalt in manuscript.

**Comment 11:** “SEM” is spelled incorrectly several times in the manuscript, and it is recommended that the authors check the whole text.

**Reply 11:** We are sorry for such a silly mistake. We have carefully checked and corrected.

**Comment 12:** In subsection 3.7, it is mentioned that SSD asphalt has low temperature sensitivity, while in conclusion 4, it is mentioned that SSD has excellent temperature sensitivity, which is contradictory.

**Reply 12:** We are sorry for our unclear expressions, we have changed the corresponding description of conclusion 4.

**Comment 13:** The evaluation of the deodorizing effect of SSD asphalt in this manuscript relies only on the Odor Intensity Rating Method (OIRM), but the results of this test are not listed in the text and need to be supplemented. However, the results of this test are not listed in the text, which should be added. Moreover, the odor intensity rating method mainly comes from the personal feeling of each volunteer, which is subjective. The TG-MS test can qualitatively and quantitatively analyze the substances in asphalt fumes, and it is suggested that the authors add more test data to evaluate the deodorizing effect of deodorizers.

**Reply 13:** Thank you for your suggestion, we have added corresponding content. However, due to our technical limitations, it is difficult for us to conduct a more detailed TG-MS analysis and we hope you can understand.

**Comment 14:** The result from Fig. 9 is that the smoke inhibition rate reaches 53% when the MOS doping is 3.0% and SBS doping is 4.0%, which is not in line with the description of the authors.

**Reply 14:** Thank you for your suggestion, but we have rechecked and confirmed that there is nothing wrong with the picture content. When the SBS content changed from 3% to 4%, the smoke inhibition rate in the figure showed an obvious decrease.

**Comment 15:** The dosage of deodorant was determined without specific experimental basis, and it is recommended that the authors add the effect of different dosages on asphalt performance to arrive at the optimum dosage of deodorant.

**Reply 15:** Thanks for your suggestion, we have added the process of determining the dosage of deodorant to the manuscript.

**Comment 16:** The writing of the conclusion is too long, it is recommended that the author streamline the conclusion and refine the core points.

**Reply 16:** Thanks for your advice. We have streamlined our conclusions and refined the core points as much as possible.

**Reviewer #2:**

**Comment 1:** It seems like the aerosol portion of asphalt fumes is neglected in the proposed asphalt fume collection device, yet it takes up a major portion. Why didn’t the authors consider the aerosol portion in this step?

**Reply 1:** We gratefully appreciate for your suggestion. We admitted that we neglected the aerosol portion of asphalt fumes in the proposed asphalt fume collection device, but it is difficult for us to adopt the mass method on the collection of the aerosol portion. So, we can only characterize the quality of asphalt fume by the weight of filterable substance, and summarize the inhibitory effect of asphalt fume. Then the thermogravimetric mass spectrometry (TG-MS) was used to comprehensively analyze the inhibition effects of small molecules, polycyclic aromatic hydrocarbons and other organic volatiles.

**Comment 2:** What are the differences between the asphalt fume collection devices of the authors and others?

**Reply 2:** The device has high air tightness, and the key point is to use PTEE hydrophobic filter membrane, which has been applied to materials, medical and other industries in recent years, with excellent and stable effects. Comparative experimental data show that the effect of improved device is better. But considering that this paper focuses on the factors affecting the release of asphalt fume, there is no space to compare the data.

**Comment 3:** According to Figure 6, the mass of asphalt fumes decreased with stirring time. Did the authors consider the effect of modification process and pre-heating process on the inhibition of asphalt fumes?

**Reply 3:** We gratefully appreciate for your valuable suggestion. It’s true that during the preparation process, bitumen smoke is already in the process of release. In our study, in order to compare various influencing factors, the same preparation conditions were used as the starting conditions. So that there was no influence of modification process and preheating process on the inhibition effect of asphalt smoke, and no collection and analysis of the smoke. Thanks for the question, and we will conduct further experiments and analysis on each link in the future

**Comment 4:** The authors developed an asphalt fume collection device, how did the authors ensure the effect of this device?

**Reply 4：**Thanks for your comment, the device is strictly cleaned by cyclohexane before the test, and strictly ensure the overall air tightness. PTFE hydrophobic filter membrane has been applied in materials, medicine and other industries in recent years, and it has excellent and stable filtration effect. Through improving the adsorption material, the test device is refined and intuitive.

**Comment 5:** How did the authors control the air flow rate?

**Reply 5：**The sample pump is equipped with an air flow meter as a constant air supply device. The first section of the device is equipped with anhydrous calcium chloride and filters out the moisture in the air to ensure the constant drying of the air flow in the entire closed device.

We tried our best to improve the manuscript and made some changes in the manuscript. These changes will not influence the content and framework of the paper. And the changes we made are marked in yellow in the revised manuscript.

Once again, thank you very much for your comments and suggestions.

Yours sincerely,

Xu Yongli
