# Supplementary material for: Study on the releasing regularity of asphalt fume and its suppression technology
Source: PLoS One. 2025 Aug 4;20(8):e0329225. doi: 10.1371/journal.pone.0329225 (PMC12321099; doi:10.1371/journal.pone.0329225)
Supplement: Attachment — Submitted filename: Response_to_Reviewers_auresp_2.docx [file pone.0329225.s003.docx]

Dear Editors and Reviewers:

On behalf of my co-authors, we would like to thank you very much for giving us the opportunity to revise our manuscript. We appreciate the reviewers very much for their positive and constructive comments and suggestions on our manuscript entitled "Study on the Releasing Regularity of Asphalt Fume and Its Suppression Technology". (Manuscript ID: PONE-D-25-06131). We have studied the comments carefully and made revisions which we hope meet with approval.

The main corrections in the paper and the responses to the reviewer's comments are as folows:

Response to the reviewers' comments:

**Reviewer #2:**

The manuscript is revised according to my comments, and the revision is satisfactory and suitable for the publication.

**Reply:** Thank you very much for your recognition, it is a great encouragement for us.

**Reviewer #3:**

In this manuscript, the release characteristics of asphalt fumes and their suppression techniques were analyzed. Modified asphalt with smoke suppression and deodorization functions were prepared by adding SBS, MOS and deodorant to the base asphalt. In addition, the asphalt under the action of smoke suppressants and deodorants was systematically studied by various methods, and its performance in smoke suppression and deodorization was discussed in detail. Generally, there are no major issues with the manuscript, but it can be improved in the following aspects.

**Comment 1:** The sources are considered old in terms of history and not the scientific content. It is better to add some modern sources.

**Reply 1:** We gratefully appreciate for your valuable suggestion. We have added relatively new references.

**Comment 2:** Please increase the resolution of the images installed in the search.

**Reply 2:** We have increased the resolution of the images, thanks again for your comments.

**Comment 3:** The conclusion is a little long and it fails to highlight the actual contribution of this study completely. It is recommended to rewrite it.

**Reply 3:** Thanks for your comments. We have revised the conclusion section.

**Reviewer #4:**

**Comment 1:** The English grammar needs to be carefully checked and improved throughout the manuscript, e.g. by a professional English language editor. The current version is difficult for readers to comprehend.

**Reply 1:** Thank you for your patience and comments. We have carefully checked the entire manuscript and corrected grammar errors, as well as modified some sentences to make them more in line with English habits.

**Comment 2:** In the test section, the flowchart may be included to clearly demonstrate the research scheme of the paper.

**Reply 2:** Thanks for your valuable advice. We have add a flowchart in 2.3.7.

**Comment 3:** It is recommended to use the same range for the vertical axis of all figures in Figure 18 ~ Figure 20.

**Reply 3:** Thanks for your comment. We absolutely understand why you recommended to use the same range for the vertical axis and we did try to make it. However, the difference in the range of the vertical axis in the Figure 18 ~ Figure 20 is quite large. If we use the same range for them, it would be hard to show the change of ionic current intensity for readers, which contradicts our original intention. For example, if you change the vertical axis of second picture in Figure 18 to the same as that of fist picture, the change of ionic current intensity of SSD would be hard to read. Therefore, after careful consideration, we believe that the current image should be retained.

**Comment 4:** To guarantee the accuracy of smoke testing, does the base asphalt utilized in the preparation of modified asphalt share the same heating history?

**Reply 4：**Thanks for your comment. The base asphalt shares the same heating history and we have added relevant explanations in the manuscript.

**Comment 5:** Kindly ensure that the paper adheres to the appropriate format and line - spacing requirements. Have the line numbers been accurately assigned?

**Reply 5：**Thank you for your suggestions. We have modified the format according to the requirements on the official website.

We tried our best to improve the manuscript and made some changes in the manuscript. These changes will not influence the content and framework of the paper. And the changes we made are marked in yellow in the revised manuscript.

Once again, thank you very much for your comments and suggestions.

Yours sincerely,

Xu Yongli
